# Hydrodynamic stress in orbitally shaken bioreactors

**DOI:** 10.1186/1753-6561-5-S8-P39

**Published:** 2011-11-22

**Authors:** Stéphanie Tissot, Martino Reclari, Samuel Quinodoz, Matthieu Dreyer, Dominique T Monteil, Lucia Baldi, David L Hacker, Mohamed Farhat, Marco Discacciati, Alfio Quarteroni, Florian M Wurm

**Affiliations:** 1 Laboratory of Cellular Biotechnology, Faculty of Life Sciences, Ecole Polytechnique Fédérale de Lausanne; 2 Laboratory of Hydraulic Machines, School of Engineering, Ecole Polytechnique Fédérale de Lausanne; 3 Chair of Modeling and Scientific Computing, School of Basic Sciences, Ecole Polytechnique Fédérale de Lausanne, 1015 Lausanne, Switzerland

## Background

Orbitally shaken bioreactors (OSRs) of nominal volume from 50 mL to 2’000 L have been developed for the cultivation of suspension-adapted mammalian cells. Here we study the hydrodynamics of OSRs for mammalian cells. The results are expected to allow the determination of key parameters for cell cultivation conditions and will facilitate the scale-up of OSRs.

## Materials and methods

CHO-DG44 cells were cultivated in suspension in 1-L bottles as described in [1]. To determine conditions under which the shear stress was harmful for the cells, the bottles were orbitally shaken on an ES-X platform (Kühner AG, Birsfelden, Switzerland) at agitation rates from 150 to 200 rpm for 24 h. Control cultures were run in parallel with agitation at 110 rpm. The velocity fields, shear stress, and free surface of a 1-L bottle at 110 rpm were simulated with Computational Fluid Dynamics (CFD). The Navier-Stokes equation was approximated with the finite element method. The simulations were all based on a mesh containing 50’000 tetrahedra and 10’000 vertices. The area of a finite element was 9.8 cm^2^. Because of the chosen discretization, each time step required the resolution of a linear system composed of 190’000 unknowns.

## Results

**Figure 1:**
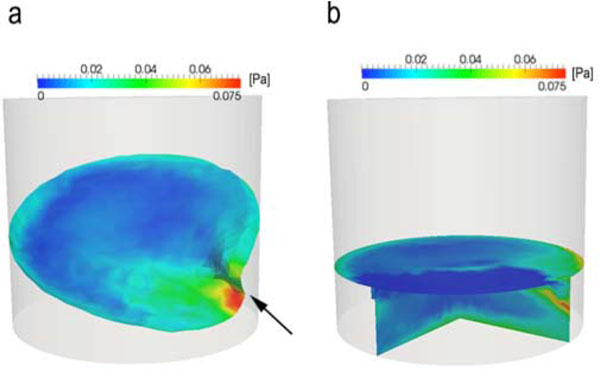
The shear stress in a 1-L bottle at 110 rpm was simulated by CFD at the free surface (a) and in the liquid phase (cut view) (b). The shaking diameter was 5 cm and the working volume was 100 mL. The arrow indicates the tip of the wave.

According to the CFD simulations of a 1-L bottle at 110 rpm, the maximal shear stress value was situated at the tip of the wave and was about 0.075 Pa (Figure 1a). The shear stress was higher at the walls than in the center of the liquid (Figure1b). Most of the zones in the liquid phase had a shear stress value equal to or lower than 0.02 Pa (Figure1b). The zones showing the maximal shear stress values represented less than 1% of the liquid phase (Figure1b).

The cell growth rate was similar for the CHO culture agitated at 110 rpm and those at 150 or 160 rpm. According to CFD simulations, the maximal shear stress was ≤ 0.17 Pa at these higher agitation rates. At 170 rpm, the cell growth rate decreased and cell damage was observed. The maximal shear stress value at this agitation rate was about 0.19 Pa. The maximal values of shear stress were always situated at the tip of the wave independently of the agitation rate. The maximal value of shear stress increased with the agitation rate. However, only a small number of zones had these maximal values.

## Conclusions

The maximal shear stress value in a 1-L OSR agitated at 110 rpm with a working volume of 300 mL was one to two orders of magnitude lower than values reported to be harmful for CHO cells [2]. This indicates that standard cultivation conditions in OSRs are safe for sensitive mammalian cells. The maximal shear stress was located at the tip of the wave of the free surface. However, as the wave of the free surface rotates with time, many zones of the liquid will be affected by the wave tip over time. Therefore, its potential to damage cells can not be neglected. Our study suggests that different wave patterns may lead to different maximal shear stress values. Further analysis of the correlation between the shape of the wave and the maximal shear stress level are required to determine a scale-up factor for hydrodynamic stress in OSRs.
